# A Highly Similar Mathematical Model for Cerebral Blood Flow Velocity in Geriatric Patients with Suspected Cerebrovascular Disease

**DOI:** 10.1038/srep15771

**Published:** 2015-10-26

**Authors:** Bo Liu, Qi Li, Jisheng Wang, Hu Xiang, Hong Ge, Hui Wang, Peng Xie

**Affiliations:** 1 Department of Neurology, The Third Hospital of Mianyang, Mianyang, Sichuan 621000, China; 2 Department of Neurology, Yongchuan Hospital, Chongqing Medical University, Chongqing 402160, China; 3 Department of Neurology, The First Affiliated Hospital of Chongqing Medical University, Chongqing 400016, China; 4 Institute of Neuroscience, Chongqing Medical University, Chongqing 400016, China; 5 Chongqing Key Laboratory of Neurobiology, Chongqing Medical University, Chongqing 400016, China

## Abstract

Cerebral blood flow velocity(CBFV) is an important parameter for study of cerebral hemodynamics. However, a simple and highly similar mathematical model has not yet been established for analyzing CBFV. To alleviate this issue, through TCD examination in 100 geriatric patients with suspected cerebrovascular disease (46 males and 54 females), we established a representative eighth-order Fourier function V_x_(t) that simulates the CBFV. The measured TCD waveforms were compared to those derived from V_x_(t), an illustrative Kolmogorov-Smirnov test was employed to determine the validity. The results showed that the TCD waves could been reconstructed for patients with different CBFVs by implementing their variable heart rates and the formulated maximum/minimum of V_x_(t). Comparisons between derived and measured TCD waveforms suggest that the two waveforms are very similar. The results confirm that CBFV can be well-modeled through an eighth-order Fourier function. This function V_x_(t) can be used extensively for a prospective study of cerebral hemodynamics in geriatric patients with suspected cerebrovascular disease.

Cerebrovascular disease has become the second leading cause of death and the leading cause of adult disability worldwide^1^,^2^. Previous studies have confirmed its close relation with the changes in hemodynamics of the cerebral vessels^3^,^4^. Functions for the cerebral blood flow velocity(CBFV) are primarily essential for study of cerebral hemodynamics^5^,^6^. The accuracy of accustomed and suitable functions substantially influences the reliability of the follow-up research results.

However, a simple and highly similar mathematical model for CBFV has not yet been established. Olufsen and colleagues^7^ reproduced the full curve of middle cerebral artery blood flow velocity on a beat-by-beat basis as modeled by a windkessel with resistors and a capacitor, but the system must input arterial pressure changes. Ursino *et al.*^8^ discussed mathematical models of cerebral blood flow regulation which derives from the interaction and superimposition of several concomitant effects. Previous studies on the cerebrovascular Computational Fluid Dynamics (CFD) chose peripheral vascular blood flow velocity functions as an alternative method for investigating the cerebral blood flow^9^,^10^. Nevertheless, these functions are too regular to completely apply to CBFV, and could lead to inevitable potential calculation errors. In our previous study, we had successfully established an eighth-order Fourier function which highly fitted cerebral perfusion pressure (CPP) in geriatric patients with suspected cerebrovascular disease^11^. Since both CBFV and CPP are periodic waves based on similar mathematical methods, we set up this study to investigate whether the mathematical model could be used to simulate the CBFV in geriatric patients with suspected cerebrovascular disease.

## Methods

The study was approved by the Ethics Committee of Chongqing Medical University. Written informed consents were obtained from all participants themselves, the methods were carried out in accordance with the approved guidelines. Our study included patients with suspected cerebrovascular disease who underwent Transcranial Doppler(TCD) examination from January 2014 to December 2014 at our institution. The inclusion criteria were as follows: 1) Patients had signs and symptoms suggestive of cerebrovascular disease. 2) Patients had to undergo TCD examinations. 3) The CBFV_max_/CBFV_min_ ranged to 30–120/15–90 cm/s, and the heart rate were within a range of 60–100 beats/min. Patients with any stenotic or occlusive disease of the carotid, vertebro-basilar and intracranial arteries along with patients acquiring arrhythmias were excluded from the study. Patients who had a recent onset of moderate to severe stroke, defined as NIHSS score > 4 points were also excluded from the study.

After attaining the TCD results, a sum of nine CBFV waves were bilaterally recorded for each patient: two from the (left and right) anterior cerebral arteries, two from the (left and right) middle cerebral arteries, two from the (left and right) posterior cerebral arteries, two from the (left and right) vertebral arteries and one from the basilar artery.

**Figure 1:**
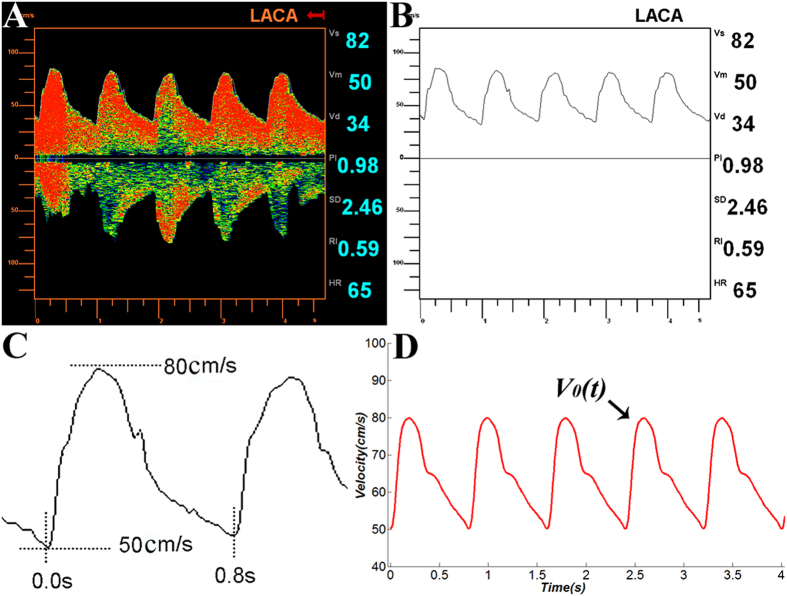
A TCD wave of the left anterior cerebral artery in a patient (A) and its black-white picture (B); standardizing a TCD wave, this procedure standardized all cardiac cycles to 0.8 s and all CBFVmax/CBFVmin to 80/50 cm/s (C);the curve of the standardized function V_0_(t) (D).

A TCD wave is an irregular periodic wave (Fig. 1A,B). Considering Parseval’s theorem, periodic signals could be summed as the superposition of all the harmonics by applying the triangular form of the Fourier series (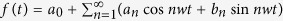, n = 1, 2, 3…)^12^. The eighth-order Fourier function 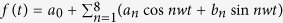 was chosen for all TCD waves separately, conferring to the function. All waves were function-fitted using MATLAB 7.0’s Curve Fitting toolbox helped fitting with the implementation of the least square method^13^,^14^. The bandwidth of the curve was set at ±3 cm/s (ordinate) such that all raw data points were located within it.

Since the TCD waves were significantly variable for all the participating patients, it was necessary to standardize all waves to derive at one representative Fourier function with 18 representative coefficients. To accomplish that, 100 patients were randomly selected followed by the standardization of all waves of these patients using the following methods: (1) the abscissa (time) was set up to 0 s at the first nadir; (2) the abscissa (time) to 0.8 s at the second nadir; (3) the ordinate of the nadir to 50 cm/s; and (4) the ordinate of the zenith to 80 cm/s (Fig. 1C). Eventually, this procedure standardized all cardiac cycles to 0.8 s and the CBFV_max_/CBFV_min_ to 80/50 cm/s.

**Figure 2:**
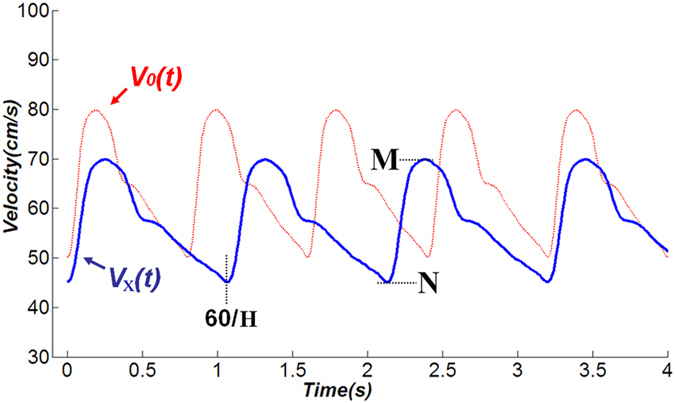
Graphical comparison of V_0_(t) and V_x_(t): the function V_x_(t) can be derived from the lengthening and/or shortening of V_0_(t) in the longitudinal and transverse directions, the V_x_(t) function can be manipulated to fit the TCD waves for differing CBFVs and heart rates.

After this standardization procedure, the 18 standardized coefficients (i.e., w, a0-a8, b1-b8) were obtained by calculating the mean of each coefficient of all new fitted functions. Based on the standardized coefficients, we were able to derive a representative eighth-order Fourier function V_0_(t) (Fig. 1D). A function V_x_(t) was derived by lengthening and/or shortening of V_0_(t) in the longitudinal and transverse directions (Fig. 2). To validate this V_x_(t) model, 100 new included patients were chosen randomly with completed TCD examination. The CBFV waves were assessed for each patient by the Fourier function model V_x_(t). Comparisons between derived and measured TCD waveforms were tested for the validity.

### Statistical Analysis

All statistical analysis was performed by using SPSS 20.0.

Definite integral of the absolute value of the differential function in [0, 1] was implemented to compare the coherent function. The single sample Kolmogorov-Smirnov test was employed to test 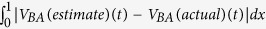, 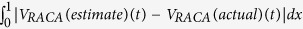, 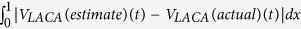, 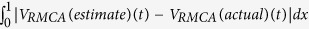, 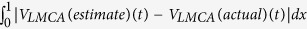, 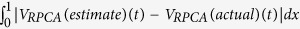, 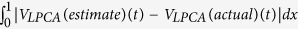, 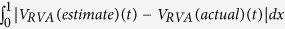, 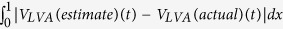 in all patients. The 95% interval <3 was considered as the same curve.

## Results

**Figure 3:**
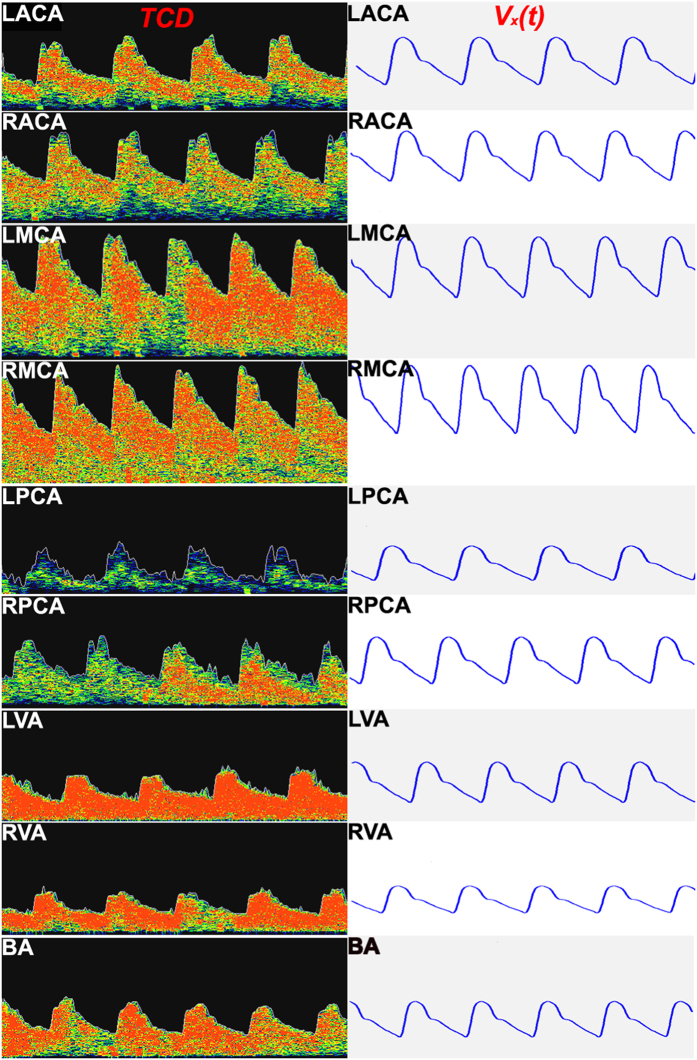
All nine TCD waves of a patient (left); estimated waveforms for each TCD wave based on the function V_x_(t) (right); comparisons between estimated and measured TCD waveforms suggest that they are very similar.

A total of 200 patients were included in our study and the demographic data was listed in Table 1. With a trend of 9 TCD waves per patients, 200 patients received a sum of 1800 TCD waves. Comparative analysis of the 9 waves received from a single patient illustrated that eventhough the 9 TCD waves were characterized with similar rhythms, they were subjected to exhibit miniature differences in the maximum and minimum. On the contrary, the gross arterial study of the 200 patients depicted that: the maximum, minimum, and rhythm of the TCD waves displayed significant differences in all patients; however, as a whole their patterns showed analogy. A typical wave starts at its nadir, then rapidly rises, and on approaching its climax follows a slower rate of speed. Some TCD waves exhibited a large angle between fast-ascent and slow-ascent phases. On approaching its culminating point, the TCD waves rapidly declined and then slowed its rate of decline until it reached its nadir (Fig. 3). During the descent phase of the TCD waves, a few waves formed a dicrotic notch, similar to the shape of the Cyrillic letter “И”.

In order to obtain the common feature of these waveforms, all 900 TCD waves belonging to the derivation group (100 patients, 49 males and 51 females, 60 to 80 years of age, each with 9 waves) were standardized with all cardiac cycles customized to 0.8 s and all CBFV_max_/CBFV_min_ to 80/50 cm/s. MATLAB 7.0 was able to construct eighth-order Fourier functions so as to fit all the standardized TCD waveforms. After the above procedure, we gained an aggregate of 900 fitting functions (100 patients, each with 9 functions) and on observation, all these new functions demonstrated as follows: these functions not only had the same maximum, minimum and rhythm, but also possessed a consistent waveform morphology; further all corresponded to a single underlying function i.e. V_0_(t) pathway.

In order to establish the standardized function V_0_(t), the 18 standardized coefficients (i.e., w, a_0_–a_8_, b_1_–b_8_) were derived by calculating the mean of each coefficient from the 900 newly fitted functions (Table 2). Based on the standardized coefficients in Table 2, we were able to derive the following representative eighth-order Fourier function: V_0_(t) = 64.5 − [5.154cos(7.85t) − 10.962sin(7.85t)] − [5.4516cos(2 * 7.85t) − 1.7574sin(2 * 7.85t)] − [1.731cos(3 * 7.85t) + 0.846sin(3 * 7.85t)] − [1.5684cos(4 * 7.85t) + 0.23976sin(4 * 7.85t)] − [0.50328cos(5 * 7.85t) + 1.0524sin(5 * 7.85t)] − [0.10014cos(6 * 7.85t) + 0.30504sin(6 * 7.85t)] − [0.035316cos(7 * 7.85t) + 0.3963sin(7 * 7.85t)] + [0.16248cos(8 * 7.85t) − 0.17118sin(8 * 7.85t)] (Fig. 1D). V_0_(t) has a cycle of 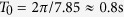, 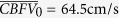, CBFV_0min_ = 50 cm/s, CBFV_0max_ = 80 cm/s.

On lengthening and/or shortening of V_0_(t) in the longitudinal and transverse directions, the V_0_(t) function was manipulated to fit the TCD waves for the varying CBFVs and heart rates (Fig. 2). Thus, in an arbitrary patient X, we could define the CBFV_max_/CBFV_min_ as M/N (cm/s) and the heart rate as H (beats/min). According to the Fourier function, based on the function V_0_(t); CBFV function V_x_(t) of this patient X, should be able to conform to the following relationship:


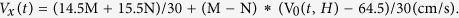


Based upon this formula, we could estimate that the V_x_(t) has an average value of (1/30)(14.5 M + 15.5 N) (cm/s), with an ordinate range from M to N, and a cycle of 60/H (s) (Fig. 2).

To validate this V_x_(t) model, a validation group (100 patients, 46 males and 54 females, 60–80 years of age) were randomly selected for TCD examinations, and later all TCD waves were function-fitted using MATLAB 7.0. The TCD waveforms were also assessed in each patient by the Fourier function model V_x_(t). The estimated TCD waveform and the actual waveform were calculated by using 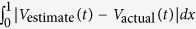. The results suggested that the two waveforms were very similar (Table 3 and Fig. 3). We further validated whether this V_x_(t) model can be used for normal subjects. A total of 100 normal subjects (51 males and 49 females, 60–80 years of age) were randomly selected for TCD examinations and all TCD waves were assessed by the Fourier function model. The single sample Kolmogorov-Smirnov test of nine definite integrals in 100 normal subjects were illustrated in Table 4. The results suggested that the estimated TCD waveform and the actual waveform were similar (Table 4).

## Discussion

Transcranial Doppler (TCD) is a noninvasive method that is commonly used for detection of the cerebral hemodynamics in patients with suspected neurovascular diseases. It is commonly used in clinical practice due to its relatively very simple operation and good reproducibility. In recent years, TCD has been widely used for measurement of cerebrovascular hemodynamics^15^,^16^. TCD raw data employ the mean blood flow velocity of the intracranial vessels, which provides important hemodynamic data. MRI might be able to detect cerebrovascular blood flow velocity, but MRI examination is relatively expensive and can not be performed continuous in patients with cerebrovascular diseases. Another advantage of TCD over MRI is that it allows non-invasive dynamic observations of cerebrovascular hemodynamic parameters in a clinical scenario^17^,^18^. TCD was adopted in this study for an objective and accurate CBFV examination and the function model V_x_(t) has successfully derived on the basis of these reliable tests.

Fourier first proposed that any periodic function could be expressed via an arithmetic sum of the constituent sine and cosine functions, which is commonly known as the Fourier series (French: Série de Fourier)^12^. In recent years, Fourier transforms have been applied extensively to numerous set-ups such as medicine, physics, number theories, combinatorial mathematics, signal processing, probability, statistics, cryptography, acoustics and optics^19^,^20^,^21^. CBFV is identical to the other parameters of the cardiovascular dynamics, with its value periodically fluctuating with the heartbeat and such fluctuations is appropriate for its use in the Fourier transforms. The Fourier function in MATLAB 7.0 software package is capable of fitting to integrating all this work. 1–7 order Fourier function curves were smooth and regular, which couldn’t be fit to the irregular TCD waves. Therefore, the 1–7 order Fourier function curves were excluded. 9-order Fourier curve fitting produced too much noise from the baseline. Consequently we chose the 8-order Fourier function for each patient’s individual TCD wave fitting function. The functions were achieved with efficient fitting results (minimum residual) and minimal noise.

We standardized all variant cardiac cycles to 0.8 s and all CBFV_max_/CBFV_min_ to 80/50 cm/s in order to obtain the common feature of these TCD waveforms. All standardized TCD waveforms shared a common feature that was very close to V_0_(t) pathway and vice versa. Therefore, the V_x_(t) function might have been beneficial only to our participants. Further studies are required to demonstrate whether this function could be used for other populations and confirm its reliability. We have selected 100 normal geriatric subjects with a quantitative assessment of sitting errors. The results suggested that the V_x_(t) function was quite similar with the actual waveform. The age group of our participants was between 60–80 years old which was the high incidence age of cerebrovascular disease^22^. Thus, V_x_(t) could be used widely in the study of cerebrovascular disease.

For the study of cerebrovascular hemodynamics in a particular patient, the CBFV data can directly make use of TCD or MRI. But these data have continuous data points and are not conducive for the subsequent calculations. Therefore, a combined use of the measured data and V_x_(t) could be utilized to achieve a double benefit with a reduced effort. In addition, while studying cerebrovascular hemodynamics, we require the function expression instead of raw data points. V_x_(t) could also be used in the study of cerebral perfusion pressure(CPP) and/or cerebral vascular resistance(CVR). CPP and CVR changes are slightly complex issues. The CBFV was increased when CPP > CVR and vice versa. The first derivative of V_x_(t) was V’_x_(t), which could directly manifest changes between CPP and CVR.

## Conclusions

Based on the TCD data, CBFV can be well-modeled through an eighth-order Fourier function using MATLAB 7.0. This function V_x_(t) can be used extensively for a prospective study of cerebral hemodynamics in geriatric patients with suspected cerebrovascular disease.

## Additional Information

**How to cite this article**: Liu, B. *et al.* A Highly Similar Mathematical Model for Cerebral Blood Flow Velocity in Geriatric Patients with Suspected Cerebrovascular Disease. *Sci. Rep.* **5**, 15771; doi: 10.1038/srep15771 (2015).

## Figures and Tables

**Table 1:** Demographics of the patients studied.

Item	Value	
	The derivation group	The validation group
Mean age_SD (range)	68_4(60–80)	68_5(60–80)
Men, n (%)	49(49)	46(46)
Women, n (%)	51(51)	54(54)
Hypertension, n (%)	94(94)	92(92)
Diabetes mellitus, n (%)	12(12)	14(14)
Hypercholesterolemia, n(%)	23(23)	21(21)
Current smokers, n (%)	15(15)	17(17)

**Table 2:** The mean value of each coefficient of 900 fitted functions after standardization.

Coefficient	Mean ± S	Coefficient	Mean ± S
a_0_	64.5 ± 3.56	w	7.85 ± 0.04
a_1_	−5.154 ± 1.44	b_1_	10.962 ± 2.95
a_2_	−5.4516 ± 1.45	b_2_	1.7574 ± 0.65
a_3_	−1.731 ± 0.27	b_3_	−0.846 ± 0.64
a_4_	−1.5684 ± 0.57	b_4_	−0.23976 ± 0.23
a_5_	−0.50328 ± 0.43	b_5_	−1.0524 ± 0.65
a_6_	−0.10014 ± 0.17	b_6_	−0.30504 ± 0.11
a_7_	−0.035316 ± 0.18	b_7_	−0.3963 ± 0.67
a_8_	0.16248 ± 0.26	b_8_	−0.17118 ± 0.48

**Table 3:** The single sample Kolmogorov-Smirnov test of nine definite integrals in 100 patients and 95% confidence interval 
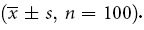

Definite integral	P-value	Mean ± S	Confidence interval(95%)	The upper boundary value
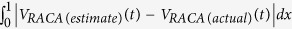	>0.05	1.63 ± 0.72	[1.53, 1.73]	<3
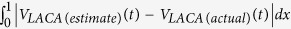	>0.05	1.55 ± 0.48	[1.45, 1.65]	<3
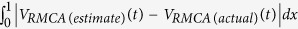	>0.05	2.05 ± 0.67	[1.94, 2.15]	<3
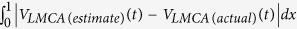	>0.05	2.00 ± 0.68	[1.91, 2.10]	<3
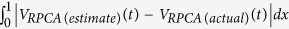	>0.05	1.44 ± 0.43	[1.37, 1.51]	<3
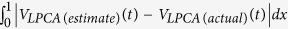	>0.05	1.65 ± 0.50	[1.58, 1.72]	<3
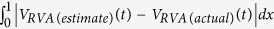	>0.05	1.12 ± 0.33	[1.06, 1.18]	<3
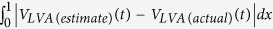	>0.05	1.07 ± 0.37	[1.0, 1.14]	<3
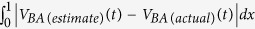	>0.05	1.12 ± 0.36	[1.04, 1.20]	<3

**Table 4:** The single sample Kolmogorov-Smirnov test of nine definite integrals in 100 normal subjects and 95% confidence interval 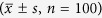.

Definite integral	P-value	Mean ± S	Confidence interval(95%)	The upper boundary value
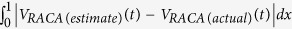	>0.05	2.88 ± 0.63	[2.74, 2.99]	<3
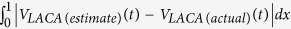	>0.05	2.70 ± 0.72	[2.55, 2.83]	<3
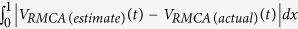	>0.05	2.69 ± 0.74	[2.54, 2.83]	<3
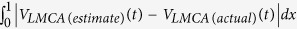	>0.05	2.75 ± 0.67	[2.62, 2.88]	<3
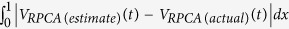	>0.05	2.70 ± 0.64	[2.57, 2.81]	<3
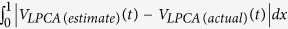	>0.05	2.73 ± 0.75	[2.58, 2.88]	<3
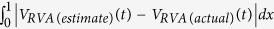	>0.05	2.59 ± 0.64	[2.45, 2.72]	<3
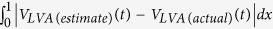	>0.05	2.52 ± 0.63	[2.39, 2.64]	<3
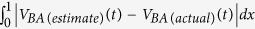	>0.05	2.61 ± 0.70	[2.47, 2.75]	<3
